# Medical Comorbidities in 181 Patients With Bipolar Disorder *vs*. Schizophrenia and Related Psychotic Disorders: Findings From a Single-Center, Retrospective Study From an Acute Inpatients Psychiatric Unit

**DOI:** 10.3389/fpsyt.2021.702789

**Published:** 2021-10-01

**Authors:** Antonella Mariano, Giorgio Di Lorenzo, Tommaso B. Jannini, Riccardo Santini, Emanuela Bertinelli, Alberto Siracusano, Cinzia Niolu

**Affiliations:** ^1^Department of Systems Medicine, Chair of Psychiatry, University of Rome Tor Vergata, Rome, Italy; ^2^IRCCS – Fondazione Santa Lucia, Rome, Italy

**Keywords:** bipolar disorder, schizophrenia, cardiovascular diseases, dysmetabolic diseases, viral diseases, medical comorbidities

## Abstract

**Introduction:** Medical comorbidities (MCs) represent a significant burden in terms of more frequent hospitalizations and overall lower life expectancy among people with severe mental disorders, such as schizophrenia and related psychotic disorders (SZ) and bipolar disorder (BD). The present article aims to compare the prevalence of MCs and to examine the associated characteristics as marital status, job occupation, level of education, and living arrangements, between BD and SZ patients.

**Methods:** One-hundred-eight-one patients with MCs (85/47% had BD and 96/53% had SZ) were recruited retrospectively from the Acute Inpatients Psychiatry Unit of Policlinico Tor Vergata, Rome, between January-2017 and December-2020. MCs were: cardiovascular diseases (CVD), bacterial infections, mycoses, viral diseases, neoplasms, musculoskeletal, respiratory tract, urological and male genital, gynecological, neurological, gastrointestinal, metabolic syndrome, nutritional, and metabolic diseases.

**Results:** BD had more MC than SZ (36.2 vs. 28.2%, respectively, *p* = 0.04). CVD and metabolic MC were more common among BD (51.8 vs.34.4%; 51.8 vs.35.3%; *p* = 0.018; *p* = 0.039; respectively), while viral diseases were more frequent in SZ (13.5 vs.3.5%, *p* = 0.035). Hypertension was common in both psychiatric illnesses (81.8% BD vs. 65.6% SZ, *p* = 0.18). Obesity was the most frequent metabolic disease in both BD and SZ (75% BD vs. 73.5% SZ, *p* = 0.91), followed by diabetes mellitus (52.3% BD *vs*. 55.9% SZ, *p* = 0.93), metabolic syndrome (54.5% BD vs. 47.1% SZ, *p* = 0.67) and dysthyroidism (47.7% BD vs. 25.7% SZ, *p* = 0.093). After performing a binary logistic regression analysis, only two MCs showed a statistically significant association: patients with SZ had an OR of 2.01 [CI 95% (1.00–4.01)] for CVD compared to BD; on the other hand, patients with BD had an OR of 16.57 [CI 95% (3.58–76.77)] for gynecological diseases compared to SZ patients.

**Conclusions:** MCs are common among people with severe mental illness, especially CVD and metabolic diseases, highlighting the need for a more collaborative relationship between general medical providers and psychiatrists.

## Introduction

Medical comorbidity (MC) is defined as the co-occurrence of multiple diseases within the same person, at the same time, regardless of the causality that links them (1). People with severe mental illness, such as bipolar disorder (BD) or schizophrenia and related psychotic disorders (SZ), have a higher prevalence of MC and a greater mortality rate, losing up to 4 years of life compared to the general population. Furthermore, the combination of mental and medical disorders is linked to an increased functional impairment, greater symptom burden, and, eventually, to a higher weight on healthcare costs (2).

A wide range of risk factors may concur to cause MCs. These might mostly be ascribed to psychiatric medications side effects (mood stabilizers, antidepressants, antipsychotics), lifestyle choices (diet, smoking, alcohol, substance abuse, exercise), and social marginalization (3). Furthermore, a consistent number of authors focused on a metabolic-inflammatory-mood pathway, suggesting that an immune system dysfunction may increase the morbidity and mortality of these patients (4). In particular, reduced levels of glutathione and antioxidant defense have been observed in first-onset psychosis (5).

Recent studies demonstrate also an interesting link between MCs and the complex and dynamical set of microorganisms, collectively referred to as the microbiota. Microbiota can have a pathogenic role in psychiatric disorders because of its modulatory action in the bidirectional communication system between the brain and intestine (widely known as the gut-brain-microbiota axis) (6). Although, there are no studies that analyze possible alterations of the microbiota during a manic episode, there is some evidence showing an altered permeability of the gastrointestinal barrier (7, 8), which can cause compositional variations of the intestinal microbiota.

Kessler et al. reported that the co-occurrence of medical and mental disorders has to be considered as the rule rather than the exception, presenting significant clinical and public health concerns (9). Similarly, Hert et al. assumed that different physical conditions are observed with increased frequency, such as stroke, hypertension, obesity, diabetes mellitus (DM), human immunodeficiency virus (HIV), and hepatitis (2). Patients with SZ are significantly more prone to have at least one MC compared to the general population, ranging from hypothyroidism to chronic obstructive pulmonary disease, diabetes, viral diseases, fluid/electrolyte disorders, and nicotine abuse/dependence (10). On the other hand, Sinha et al. in their comprehensive review found a high prevalence of MCs involving multiple organs in patients with BD, suggesting screening each patient for the added risk of correlated diseases like migraine and asthma (11).

Since MCs represent a significant burden in terms of more frequent hospitalizations and overall lower life expectancy (2), there is an urgent need to better define such correlation in patients with severe mental illnesses. For this reason, the present article aims to compare the prevalence of MC between BD and SZ and examine the associated characteristics such as social status, job occupation, level of education, and living status.

## Materials and Methods

This was a single-center, double-arm, retrospective cohort study. Nine hundred and sixty-five patients were admitted to the tertiary care center for treatment of the psychiatric disorder (Psychiatry Unit, Policlinico Tor Vergata, Rome, Italy) between January 2017 (the starting day of inpatient acute psychiatric service) and December 2020. Inclusion criteria were: diagnosis of BD or SZ [according to the International Classification of Disease, 9th Revision (ICD-9) (12)] and presence of MCs. Exclusion criteria were: comorbidity of psychiatric disorders and pregnancy. Diagnoses of MCs were made using the ICD-9. The presence of metabolic syndrome (MetS) was considered only if defined according to The US National Cholesterol Education Programme Adult Treatment Panel III (ATP III), meaning of at least three of the following criteria: waist size of at least 102 cm for men and at least 88 cm for women; triglycerides of at least 150 mg/dl; HDL cholesterol level of <40 mg/dl for men and <50 mg/dl for women; blood pressure of more than 130 mmHg systolic or 85 mmHg diastolic fasting glucose of more than 100 mg/dl (13).

Patients who did more than one recovery within the study timeframe were considered unitary. 181/965 (18.8%) patients met the inclusion criteria and were divided into two arms: 85/181 (47%) were classified as BD and 96/181 (53%) as SZ, with at least one MC.

The data were collected by three psychiatrists at the time of the discharge from the original medical records on the medical informative system.

Baseline sociodemographic data, such as gender, age, employment status (i.e., employed -. homemakers and students - or unemployed), educational level (i.e., elementary school, middle school, high school, university), and marital status (i.e., married or cohabiting, divorced, widowed, single) were collected. Medical comorbidities, such as cardiovascular diseases (CVD), bacterial infections and mycosis, viral diseases, neoplasms, musculoskeletal diseases, respiratory tract diseases, urological and genital diseases, gynecological diseases, neurological diseases, gastrointestinal diseases, nutritional and metabolic diseases were recorded. The rate of readmission was aggregated between the two groups.

### Statistical Analysis

The Kolmogorov-Smirnov test for the goodness of fit was performed to assess the normality of the distribution of all the tested variables. Continuous normal variables were expressed as mean ± standard deviation. Continuous non-normal variables were expressed as median and 95% confidential interval (CI). Demographic and clinical characteristics were analyzed using chi-square tests with Yates' correction and Odds Ratio for categorical variables and Student's *t*-tests or Wilcoxon signed-rank test for continuous variables as appropriate. Logistic regression analyses were performed using gender, age, educational level, employment status, and diagnostic group (BD and SZ) as covariates; the dependent variables were MCs. Statistical analysis was performed using MedCalc 18.2.1 software (MedCalc, Mariakerke, Belgium). *P* < 0.05 were considered statistically significant, and all *p*-values were calculated using a two-tailed significance level.

## Results

181/965 (18.8%) patients (age mean: 49 ± 13 years; women: 51.4%) met inclusion criteria and were divided into two arms: 85/181 (47%) BD with MCs (age mean: 52.2 ± 13 years; women: 57.7%) and 96/181 (53%) SZ with MC (age mean: 45.6 ± 13 years; women: 45.8%).

### Sociodemographic Characteristic

Parental-family living was more common in SZ rather than BD (51.0 vs.21.2%; *p* < 0.001), like also unemployment (90.6 vs.69.4.2%; *p* = 0.0006) and single-marital status (64.5 vs.30.6%; *p* < 0.001). Regarding the educational level, most people with BD had higher school license (40%), while in people affected by SZ middle school license was more prevalent (55.2%). The complete sociodemographic data on the 181 patients was given in Table 1.

**Table 1:** Sociodemographic information of the sample.

	**BD** **(*n* = 85)**	**SZ** **(*n* = 96)**	**Statistics**		
			**Chi-square** ^*^	***p-*value** ^**^	**Odds Ratio (CI 95%)**
*Age (SD)*	52.2 (13.0)	45.6 (13.0)			
*Gender*					
Women	49 (57.7%)	44 (45.8%)	1.12	0.29	1.6 (0.91–2.95)
Men	36 (42.4%)	52 (54.2%)			
*Employment status*					
Currently employed	26 (30.6%)	9 (9.4%)	11.68	*0.0006*	4.26 (1.86–9.74)
Not currently employed	59 (69.4%)	87 (90.6%)			
*Marital Status*					
Single	26 (30.6%)	62 (64.6%)	19.52	*<0.0001*	0.24 (0.13–0.49)
Married/Cohabiting	38 (44.7%)	18 (18.8%)	9.9	*0.016*	3.5 (1.80–6.83)
Divorced	15 (17.6%)	15 (15.6%)	0.03	0.87	1.16 (0.53–2.54)
Widowed	6 (7.1%)	1 (1%)	2.92	0.087	7.22 (0.85–61.2)
*Living arrangements*					
Independent	25 (29.4%)	29 (30.2%)	0.002	0.96	0.96 (0.51–1.82)
Living with parents	18 (21.2%)	49 (51.1%)	15.99	*0.0001*	0.26 (0.13–0.50)
Living with own family	42 (49.4%)	17 (17.7%)	17.77	*<0.0001*	4.54 (2.31–8.91)
*Educational level*					
Elementary school	7 (8.2%)	12 (12.5%)	0.48	0.49	0.63 (0.24–1.68)
Middle school	32 (37.6%)	53 (55.2%)	4.9	0.027	0.49 (0.27–0.89)
High school	34 (40.1%)	26 (27.1%)	2.84	0.092	1.80 (0.96–3.35)
University	12 (14.1%)	5 (5.2%)	3.22^**^	0.073	2.99 (1.01–8.88)

* *degree of freedom = 1*.

** *Yates correction was applied due to the low numerosity of the sample size*.

### Medical Comorbidities

**Figure 1:**
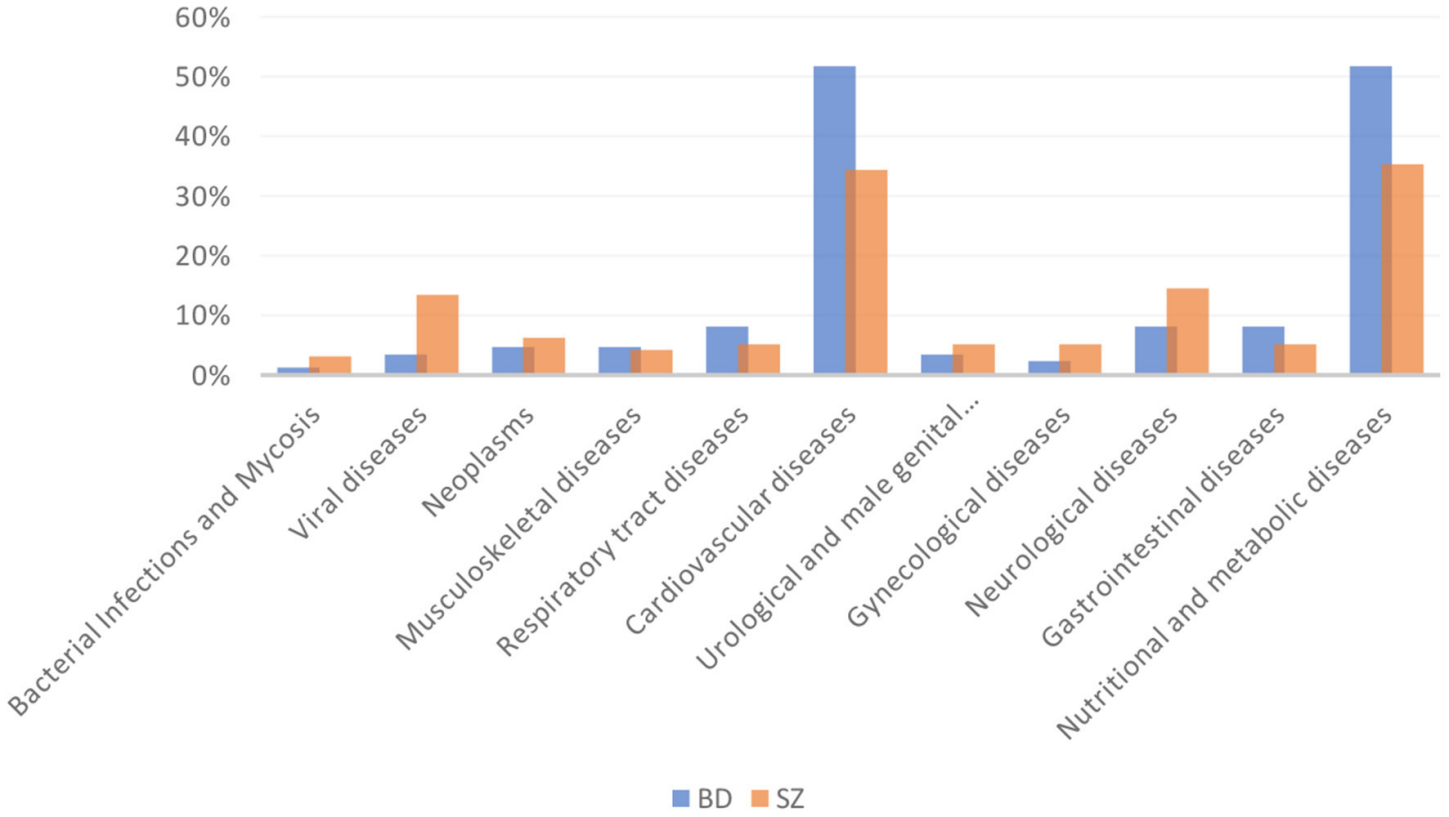
Percentage of medical comorbidities in BD and SZ.

BD patients had an overall higher prevalence of MCs compared to SZ (36.2 vs.28.2%, respectively, *p* = 0.04). In particular, CVD and metabolic diseases were more frequent among BD than SZ (51.8 vs.34.4%; 51.8 vs.35.4%; *p* = 0.018; *p* = 0.039; respectively). The most common CVD in both psychiatric illnesses was hypertension (defined by a clinic systolic blood pressure >140 mmHg or diastolic blood pressure >90 mmHg) (36/44 [81.8%] BD vs. 21/33 [63.6%] SZ). The prevalence of viral diseases was higher among SZ than BD (13.5 vs.3.5%, *p* = 0.035). Among nutritional and metabolic diseases, obesity was the most frequent disease in both BD and SZ (75% BD vs. 73.5% SZ), followed by DM (52.3%BD vs. 55.9% SZ), MetS (54.5% BD vs. 47.1% SZ), and dysthyroidism (47.7% BD vs. 25.7% SZ). No significant differences were found among the other MCs between the two arms of the study. The prevalence of MCs by category is shown in Table 2 and Figure 1. The incidence of specific medical illnesses is shown in Table 3.

**Table 2:** Number of medical comorbidities in BD and SZ.

	**BD**	**SZ**	**Statistics**		
			**Chi-square** ^*^	***p*-value**	**Odds Ratio (CI 95%)**
Bacterial Infections and Mycosis	1 (1.2%)	3 (3.1%)	0.15	0.70	0.37 (0.038–3.62)
Viral diseases	3 (3.5%)	13 (13.5%)	4.43	*0.035*	0.23 (0.064–0.85)
Neoplasms	4 (4.7%)	6 (6.3%)	0.016	0.90	0.74 (0.20–2.72)
Musculoskeletal diseases	4 (4.7%)	4 (4.2%)	0.035	0.85	1.14 (0.28–4.69)
Respiratory tract diseases	7 (8.2%)	5 (5.2%)	0.27	0.61	1.63 (0.50–5.35)
Cardiovascular diseases	44 (51.8%)	33 (34.4%)	5.55	*0.018*	2.05 (1.13–3.73)
Urological and genital diseases	3 (3.5%)	5 (5.2%)	0.035	0.85	0.67 (0.15–2.87)
Gynecological diseases	2 (2.4%)	5 (5.2%)	0.37	0.54	0.44 (0.083–2.32)
Neurological diseases	7 (8.2%)	14 (14.6%)	1.21	0.27	0.52 (0.20–1.37)
Gastrointestinal diseases	7 (8.2%)	5 (5.2%)	0.27	0.61	1.63 (0.50–5.35)
Nutritional and metabolic diseases	44 (51.8%)	34 (35.4%)	4.27	*0.039*	1.96 *(1,08–3,55)*

* *degree of freedom = 1*.

**Table 3:** Incidence of specific medical illnesses.

	**BD**	**SZ**
Hypertension	36 (42.4%)	21 (21.9%)
Obesity	33 (38.8%)	25 (26%)
Diabetes	23 (27.1%)	19 (19.8%)
Metabolic syndrome	24 (28.2%)	16 (16.7%)
Dysthyroidism	21 (24.7%)	9 (9.4%)
HIV	2 (2.4%)	5 (5.2%)

After performing a binary logistic regression analysis, using age, gender, educational level, employment status, and diagnostic group (BD or SZ) as covariates, only two MCs showed a statistically significant association. In particular, patients with SZ had an OR of 2.01 [CI 95% (1.00–4.01)] for CVD compared to BD; on the other hand, patients with BD had an OR of 16.57 [CI 95% (3.58–76.77)] for gynecological diseases compared to SZ patients. Results by category are shown in Table 4.

**Table 4:** Logistic regression results.

	**BD coefficient**	**SZ coefficient**	**Statistics**	
			***p*-value**	**Odds Ratio (CI 95%)**
Bacterial Infections and Mycosis	0.16	–	0.90	1.18 (0.08–16.84)
Viral diseases	1.09	–	0.14	2.98 (0.71–12.57)
Neoplasms	0.045	–	0.95	1.05 (0.22–5.05)
Musculoskeletal diseases	0.0022	–	1.00	1.023 (0.21–4.75)
Respiratory tract diseases	–	0.53	0.43	1.69 (0.46–6.20)
Cardiovascular diseases	–	0.70	0.049	2.01 (1.00–4.01)
Urological and genital diseases	0.94	–	0.29	2.57 (0.45–14.77)
Gynecological diseases	2.81	–	0.0003	16.57 (3.58–76.77)
Neurological diseases	0.30	–	0.59	1.35 (0.45–4.03)
Gastrointestinal diseases	–	0.42	0.57	1.53 (0.36–6.57)
Nutritional and metabolic diseases	0.50	–	0.14	0.61 (0.32–1.18)

## Discussion

Parental leaving, unemployment and single-marital status were more common among SZ than BD patients (51.0 vs.21.2%; 90.6 vs.69.4.2%; 64.5 vs.30.6%). Impairment for SZ patients in areas of intimate relationships, occupational activity, and living situations was described in different other studies (14, 15).

The present study findings are consistent with the results of Brekke, which reported that people with SZ are having impairment in vocational ability, lack of adequate social competence, and necessary social skills in the workplace (16).

On the contrary, the episodic course of illness combined with above-average educational skills would explain why patients with BD were more motivated to seek treatment, as well as more able to engage in the treatment process compared to SZ individuals (17). This results in a stronger incentive to maintain contact with the services and therefore achieve a better quality of life.

The prevalence of overall MCs among people with BD (36.2% BD vs. 28.2% SZ, *p* = 0.04) was slightly inferior compared to those from prior studies, whose ranges were of almost 40–50%, with CVD, metabolic, respiratory, and musculoskeletal diseases being the medical categories mostly involved (18, 19). Although people with mental disorders have a higher percentage of risk factors for cancer (smoking, obesity, reduced physical activities), some studies have shown the paradoxical finding that, apparently, they also have a lower risk of cancer (20).

This numerical divergence could emerge from the differences in exclusion criteria and the study design. In particular, this might be related to the comparison between acute inpatients and chronic lithium-dependent outpatients and between observational and randomized controlled trials (18).

A reason for explaining the higher prevalence of MCs in BD compared to SZ patients could be found, other than the already known adverse effects of unhealthy lifestyle and psychotropic medications, in a new link between common MCs and pathological pathways in BD. Studies have shown that in BD inflammation and oxidative stress pathways might play a key role in the co-occurrence of neuroprogression and MCs (21, 22).

Furthermore, patients with SZ are very often affected by thought disorders and executive dysfunctions, so that they become unable to look after their own health. This weighs not only on their psychological conditions but also causes them to procrastinate the diagnosis of their “hidden” medical illnesses (3).

As previously shown by other studies, CVD was the most common MCs among both BD and SZ patients (51.8 vs.34.4%; *p* = 0.001). This may be explained by the fact that, in addition to the conventional CVD risk factors (i.e., obesity, smoking, raised blood cholesterol), other predisposing factors, such as physical inactivity, unhealthy diet, and low socioeconomic status, are very common among these patients.

Evidence from previous studies reported increased rates of “any cardiovascular illness” such as angina, stroke, myocardial infarction, hypertension, in patients with BD (23–25). For instance, McEvoy et al. found out that coronaropathy was 2–3.6-fold higher in patients with SZ (26), while cerebrovascular diseases seemed to be 2.1–3.3-fold higher in patients with BD compared to the general population (27, 28). To this end, it is easy to understand how almost 25% of patients with BD and 33% of patients with SZ die for CVD (29).

In line with this, Puntervold et al. showed in an autoptic study the prevalence of undiagnosed diseases, with cardiovascular illnesses being more frequent in SZ patients compared to controls (30).

The authors pointed out how people with such psychiatric conditions had difficulties in taking care of themselves and potentially explaining the higher prevalence of diagnosed MC in BD in the present series.

Among CVD, hypertension was the most frequent disease in both BD and SZ (36/44 [81.8%] vs. 21/33 [63.6%]). This finding is in line with previous studies showing the prevalence of CVD in severe mental illnesses ranging 35%-61% in BD vs. 19%-58% in SZ (31, 32).

In a very large study involving 25,339 people with BD and a control population of 113,698 individuals, Johannessen et al. showed an increased rate of hypertension among those with BD compared with both the control population and subjects with SZ (33). Interestingly, the authors highlighted how medications might surely have impacted the development of hypertension. However, the prevalence of MCs was also to be correlated to an unhealthy lifestyle and high levels of sedentary behavior, abuse of substance or alcohol, and mental health symptoms such as memory impairment, difficulties in taking appointments with the doctor, reduce motivation. Furthermore, clinicians often had wrong beliefs about these people, underestimating their capabilities of taking care of themselves (34). Regarding this, Chen et al. found out that people suffering from SZ, who were employed or lived in a highly urbanized area, had a decreased risk of sudden cardiac death (35).

For these reasons, a more consistent screening for those with BD and SZ should be implemented as quickly as possible, in order to prevent worse cardiovascular scenarios from occurring and therefore to reduce the overall mortality.

In the present series, patients with BD have a higher prevalence of endocrine, nutritional and metabolic diseases (51.8 vs.35.4%; *p* = 0.039). Consistent with our findings, literature reports how obesity, DM, MetS, and dysthyroidism may be associated with multiple risk factors, including unhealthy lifestyles (36, 37) psychotropic medications (particularly atypical antipsychotics, affecting between 15 and 72% of patients) (38–40), and abuse of recreational drugs.

When it comes to BD, different studies suggest that these patients tend to be more overweight than people with SZ and consequently have a higher risk of MCs, only because of their medical treatment (e.g., lithium, anticonvulsants, *etc*.) but also the possible comorbid binge-eating disorders, depressive episodes, low exercise and a diet full of carbohydrate (41–43). In this regard, more recent works show that patients with elevated Body Mass Index (BMI) have been associated with more frequent and longer depressive episodes (42, 44), poor response to treatment suicidal ideation, and a history of suicide attempts (45, 46) comorbid anxiety disorders (44, 47), and functional impairment (47, 48).

Obesity is also associated with MetS, which confers a 5- to 6-fold increased risk of developing DM and a 3- to 6-fold increased risk of coronary heart diseases. Among SZ, percentages vary between 19.4 and 68%, depending on age, gender, MetS criteria, and ethnicity (2) Mitchell et al., in a meta-analysis of 77 publications, showed a MetS prevalence of 32.5% among adults with SZ (49).

Furthermore, different studies regarding MetS and BD, reported a percentage of 22–30% (50–52). Comparing the data obtained with the Italian literature, no statistically significant differences were reported. Rossi et al. reported MetS prevalence rates in subjects with BD and SZ of 36.4% and 30.6%, respectively (53). Similarly, Maina et al. highlighted the presence of MetS in 27.9% in 185 patients with BD (54).

Important in the prevalence of Mets are lifestyle and behavioral patterns, such as cigarette smoking, substance abuse, physical inactivity, and overeating, which lead to insulin resistance and, frequently, in overweight. Also, specific psychotropic medications (increasing MetS dysregulations) and genetic vulnerability, and pathophysiological mechanisms play a role in MetS. In fact, in a study carried out by Maina et al. on 70 subjects with BD, a significant increase in the MetS prevalence, over a period of 2 years, from 28.6 to 44.3%, was found out, especially in patients treated with atypical antipsychotics (55).

Regarding diabetes mellitus, both in people with BD and with SZ, the prevalence is 2- to 3-fold higher compared to the general population (56, 57). The reason for the increased risk of DM in these patients is multifactorial and includes medical treatment, genetic factors, and lifestyle. Among psychotropic medications, in addition to second-generation antipsychotics olanzapine and clozapine (58, 59), lithium and valproic acid are known to induce weight gain and dysregulation in glucose metabolism (43, 60).

Furthermore, less than half of people with BD and DM in the National Health and Nutrition Examination Survey were able to reach the glycemic control goal, considering that BD patients have high rates of treatment non-adherence and recurrence (61).

In respect to thyroid diseases, our sample is in line with findings reported in the literature, showing a higher prevalence of thyroid hypofunction among patients with BD than patients with SZ. The prevalence of dysthyroidism among people with BD might surely be related to lithium's direct antithyroid effects, whose prevalence ranges from 14 to 17 % (62). For instance, Joffe et al. evaluated 42 patients with BD who received at least 3 months of lithium treatment: 19% of them required thyroid replacement or showed evidence of subclinical hypothyroidism (63). As Lambert et al. highlighted, the average 4-year risk of developing hypothyroidism was higher for lithium (8.8%) compared to quetiapine (8.3%), lamotrigine (7.1%), valproate (7.02%), aripiprazole (7%), carbamazepine (6.7%), risperidone (6.5%), olanzapine (6.4%), and oxcarbazepine (6.3%). Nevertheless, the risk of hypothyroidism has to be considered concrete regardless of the treatment. Recent evidence shows in fact that impaired levels of thyroid hormone were evident across all BD medications, highlighting the need for a thyroid screen irrespective of the medication used (64).

In our sample patients with SZ have a high prevalence of viral diseases (13.5% SZ vs. 3.5%BD, *p* = 0.035). In particular, HIV was the most prevalent [5/13 (35.5%)], followed by hepatitis B virus [4/13 (30.8%)] and hepatitis C virus [4/13 (30.8%)]. This could be linked to the higher frequency of substance abuse, sexual risky behaviors (e.g., sex without a condom, trading sex for money and drugs), and a reduced knowledge about HIV-related safety concerns (65). Furthermore, patients with both HIV and psychiatric conditions are more at risk of developing metabolic issues, as, other than antipsychotic therapies, also antiretroviral medicines may enhance the occurrence of metabolic abnormalities and ultimately of CVD (66).

After performing the binary logistic regression analysis, only two MCs showed a statistically significant association.

CVD predicts the diagnosis of SZ (OR 2.01), independently from socio-demographic characteristics. C VD increased the mortality rate of SZ both including personal-related factors (metabolic syndrome, sedentary behavior, tobacco smoking) and drug-related factors (67), with a 90% higher mortality rate for CVD comparing with the general population (68). However, guidelines still do not advocate for a routine check of CVD in these patients (67). The present study suggests that screening for CVD in SZ may be helpful for early diagnosis and potentially for reducing mortality.

Gynecological diseases significantly predict the diagnosis of bipolar disorder (OR 16.57). Although this data may be affected by gender distribution, our spread is quite similar (female BD 57.7% *vs*. SZ 45.8%). Moreover, endometriosis and polycystic ovary syndrome (PCOS) are more frequent in BD (69).

Endometriosis affects nearly 10–15% of women and is one of the main causes of female infertility (70). As reported by Pope et al. in a review, 16.7% of the women with endometriosis met the criteria for one of the forms of bipolar disorder, compared to only 2.7% of women in the comparison group (71).

Endometriosis has been reported to have significant psychosocial implications, such as depression, increased stress, and anxiety, which can also influence its clinical evaluation and the success of interventions (71).

PCOS is a common disorder, affecting up to 15–20% of reproductive age women, which manifests as hirsutism, menstrual irregularity, subfertility (72). A large amount of literature has shown that women with PCOS have a poor quality of life (73–75) and different medical disorders such as elevated testosterone level (in the cerebrospinal fluid of SZ patients) (76), obesity (77), insulin resistance (78) and inflammation (72). Nevertheless, further research is needed to strengthen these findings.

This study suffers from some limitations—notably related to its retrospective design. Firstly, since many health professionals have contributed to the collection of medical records, patients' clinical history might have been taken with less strict criteria than those achievable with a prospective design. Secondly, the present work is a single-center study. This may bias the induction of our results to other centers. Thirdly, psychiatric comorbidities that could also weigh on the overall clinical picture, such as substance use disorder, were not considered as the study was focused only on medical comorbidities. Moreover, although some patients had more than one medical comorbidity, they were considered separately and only once in the statistical analysis to make results more understandable and applicable. Since we did not set medical variables, such as DM or nutritional and metabolic diseases, as dependent variables in the logistic regression, we cannot exclude for these latter to also predict CVDs in people with BD and SZ. Lastly, this is a cross-sectional study, and the lack of follow-up data does not allow to make any causal inferences. For these reasons, extra caution must be taken when generalizing these findings.

## Conclusions

In summary, regarding sociodemographic features, parental-family living, unemployment, and single-marital status were more common among patients with SZ. Thus, it seems essential to implement an action plan that seeks to promote the autonomy of these individuals and their insertion into society.

Overall MCs were more common among BD patients, especially cardiovascular and dysmetabolic diseases, while viral diseases had a higher prevalence among SZ patients. Both CVD and gynecological diseases predict SZ and BD, respectively. Medications, lifestyle choices (such as unhealthy diet, smoking, alcohol and substance abuse), and social marginalization could be considered as the most common risk factors. Thus, psychiatrists and primary healthcare providers should promptly identify these clinical risk factors, in order to give patients an appropriate, integrative and complete medical treatment.

## Data Availability Statement

The raw data supporting the conclusions of this article will be made available by the authors, without undue reservation.

## Author Contributions

AM: manuscript drafting, patients information collection, and statistical analysis. GD, AS, and CN: manuscript critical revision. TJ and RS: patients information collection and literature review. EB: patients information collection. All authors have read and approved the final manuscript.

## Conflict of Interest

The authors declare that the research was conducted in the absence of any commercial or financial relationships that could be construed as a potential conflict of interest.

## Publisher's Note

All claims expressed in this article are solely those of the authors and do not necessarily represent those of their affiliated organizations, or those of the publisher, the editors and the reviewers. Any product that may be evaluated in this article, or claim that may be made by its manufacturer, is not guaranteed or endorsed by the publisher.

## Abbreviations

MCs: Medical comorbidities
SZ: Schizophrenia and related psychotic disorders
BD: Bipolar disorder
CVD: Cardiovascular diseases
DM: Diabetes mellitus
MetS: Metabolic Syndrome
HIV: human immunodeficiency virus
PCOS: Polycystic ovary syndrome.
